# Multi omics network toxicology and *in vitro* experiments elucidate the role of benzo [a] pyrene in prostate cancer

**DOI:** 10.3389/fcell.2026.1768139

**Published:** 2026-03-27

**Authors:** Zhenwei Liu, Qingqing Ren, Shanchang Zhou, Guofu Liang

**Affiliations:** 1 Department of Urology, Hechi Traditional Chinese Medicine Hospital, Hechi, China; 2 Endocrinology Department, Hechi Traditional Chinese Medicine Hospital, Hechi, China

**Keywords:** benzo[a]pyrene, machine learning framework, network toxicology, prostate cancer, RRM2

## Abstract

**Background:**

In recent years, growing attention has been paid to the role of Benzo [a]pyrene (BaP) in the development and progression of prostate cancer (PCa). However, the specific molecular mechanisms remain unclear. This study aims to explore the potential association between BaP and PCa and to identify key molecular targets that may underlie this relationship, using an integrative bioinformatics approach.

**Methods:**

This study initiated with a computational toxicology assessment of BaP’s carcinogenicity and endocrine-disrupting properties using the ProTox 3.0 platform. Subsequently, potential target genes linking BaP to PCa were identified by integrating multiple public databases. The overlapping genes underwent PPI network construction and visualization, followed by GO functional annotation and KEGG pathway enrichment analyses to elucidate the underlying biological mechanisms. Through screening 101 machine learning algorithm combinations, we identified the most relevant key genes associated with PCa progression. Molecular docking technology was then employed to evaluate the binding interactions between BaP/natural active products and these key targets. The CIBERSORT algorithm was utilized to analyze RRM2’s regulatory role in the PCa tumor microenvironment, complemented by pan-cancer analysis to investigate RRM2’s universal functions across various malignancies. Finally, *in vitro* cell experiments were conducted for validation.

**Results:**

This study further underscores the carcinogenic properties and endocrine-disrupting effects of BaP. Integration of multi-source databases identified 443 potential BaP-PCa targets. GO and KEGG enrichment analyses revealed that these targets are primarily involved in regulating cell proliferation, inflammatory responses, oxidative stress, and multiple oncogenic signaling pathways. Machine learning algorithm screening showed that the Enet (α = 0.1) model exhibited the best predictive performance and robustness. Through molecular docking, Kaplan-Meier survival analysis, and validation using the Human Protein Atlas (HPA) database, RRM2 was identified as a key regulatory gene and found to play a central role in BaP-mediated immunosuppression processes. Pan-cancer analysis demonstrated that RRM2 has universal functions across various malignancies. Molecular docking results indicated that seven known anti-tumor natural products exhibit significant binding affinity with RRM2. *In vitro* experiments demonstrated that BaP treatment was associated with increased RRM2 expression in prostate cancer cells, while baicalin treatment reduced this effect, providing preliminary experimental support for the bioinformatic predictions.

**Conclusion:**

This study delineates a potential mechanistic framework by which BaP may be associated with PCa progression through multi-target and multi-pathway mechanisms, highlighting RRM2 as a candidate core mediator. These findings provide a theoretical foundation for future experimental validation and epidemiological studies.

## Background

Prostate cancer (PCa) is a clinically heterogeneous malignancy characterized by significant variability in its clinical manifestations (Wang et al., 2018). As the most frequently diagnosed malignancy and the second leading cause of cancer-related mortality among men globally, PCa poses a serious threat to public health and imposes a substantial healthcare burden (Siegel et al., 2024; Raychaudhuri et al., 2025). However, the precise molecular mechanisms underlying its high incidence remain incompletely understood. Beyond established risk factors such as age, ethnicity, and family history, the specific roles of environmental exposures, lifestyle factors, and dietary components in PCa pathogenesis require further in-depth investigation (Wang et al., 2018; Cui et al., 2024; Feijó et al., 2025).

Benzo[a]pyrene (BaP) is a classic environmental pollutant generated during the incomplete combustion of organic matter in industrial processes (Huang et al., 2025). Global exposure assessment data indicate a daily BaP intake of 50–613 ng/day in the general population, with dietary sources and air pollution significantly contributing to a cumulative cancer risk of up to 5.163 × 10^−6^ (Huang et al., 2025; Hummel et al., 2018). Furthermore, specific occupational settings can exacerbate exposure levels, with lifetime cumulative doses reaching 300 μg/m^3^·year (Araki et al., 2024). As a typical endocrine-disrupting chemical, BaP’s capacity to interfere with normal hormonal function has been clearly demonstrated (Zhang et al., 2016; Bukowska et al., 2022). Given the crucial role of hormonal signaling pathways in PCa development and progression, BaP exposure may disrupt endocrine homeostasis, thereby affecting core biological processes such as cell proliferation, differentiation, and apoptosis, ultimately promoting tumorigenesis (Feijó et al., 2025; Modica et al., 2023; Shi et al., 2017; Patke et al., 2024). Although existing studies have associated BaP exposure with various adverse health outcomes, the specific molecular mechanisms underlying its role in PCa initiation and progression await systematic elucidation.

Against this backdrop, the rapid advancement of bioinformatics provides crucial technical support for deciphering the complex mechanisms linking environmental exposures to disease occurrence (Li et al., 2025; Goldenberg et al., 2019). Network toxicology, as an emerging methodology that integrates multi-omics strategies with network analysis technologies, enables systematic exploration of the potential mechanisms through which toxicants induce disease, and has been widely applied to investigate the pathogenic mechanisms of environmental factors and identify key therapeutic targets (Goldenberg et al., 2019; Shi et al., 2024). Concurrently, machine learning algorithms have demonstrated significant advantages in disease risk prediction and core biomarker screening (Ngiam and Khor, 2019). Leveraging these methodological advances, this study integrates network toxicology, multi-omics analysis, machine learning algorithms, and molecular docking to generate hypotheses regarding the potential association between BaP exposure and PCa development, and to propose candidate molecular mechanisms that warrant further experimental investigation.

## Materials and methods

### Data sources

We integrated transcriptomic data and matched clinical information from 1,028 prostate cancer (PCa) patients, sourced from: (1) the TCGA-PRAD cohort (accessed via the UCSC Xena platform) (https://xena.ucsc.edu/), and (2) three independent cohorts from the GEO database (GSE21032, GSE70770, and GSE116918) (https://www.ncbi.nlm.nih.gov/geo/summary/). To enhance statistical power, GSE21032 and GSE70770 were merged into a combined cohort (designated the GSE cohort). To ensure data reliability, patients lacking biochemical recurrence (BCR) status or with follow-up time less than 1 month were excluded. Detailed clinical characteristics of each cohort are summarized in Supplementary Tables S1 and S2. Additionally, the Human Protein Atlas (HPA) (https://www.proteinatlas.org/) and SangerBox databases (http://sangerbox.com/home.html) were employed for protein expression validation and pan-cancer analysis, respectively.

### Toxicity prediction

ProTox 3.0 is an online computational platform for predicting the toxicity of small molecules (Ngiam and Khor, 2019). The SMILES structure of BaP, obtained from the PubChem database, was used to systematically predict its toxicity profile and related parameters on the ProTox 3.0 platform. This tool covers multiple toxicological endpoints, enabling a comprehensive assessment of BaP’s potential toxicity and safety risks, thereby providing a reference for subsequent experimental validation and risk management.

### Acquisition of BaP target genes

To comprehensively identify potential targets of BaP, we queried four authoritative databases: SwissTargetPrediction, TargetNet, PharmMapper, and the Comparative Toxicogenomics Database (CTD). The canonical SMILES string and SDF structure file of BaP were obtained from the PubChem database. The following thresholds were applied to screen for high-confidence human targets: probability >0.01 for SwissTargetPrediction, AUC ≥0.7 for TargetNet, conformations = 300 and energy cutoff = 20.0 kcal/mol for PharmMapper, and Interactions >10 for the CTD database. During data standardization, the UniProtKB database was used to uniformly correct gene symbols and functional annotations of predicted targets, with Entrez Gene ID serving as the unique identifier.

### Acquisition of PCa targets

Following previously reported methods [reference needed], transcriptomic data from 534 patients in the TCGA-PRAD cohort, comprising 483 tumor tissues and 51 normal control tissues (see Supplementary Table S2), were analyzed. Differential expression analysis was performed using the DESeq2 R package, with screening thresholds set at |log2FoldChange| > 0.5 and adjusted p-value <0.05. This threshold was selected to balance sensitivity while capturing a broader spectrum of potentially biologically relevant genes.

### Identification of BaP-PCa targets and PPI network construction

Common targets between BaP and PCa were identified using Venn analysis. Subsequently, protein-protein interaction (PPI) analysis was performed on these overlapping targets using the STRING database (confidence score threshold ≥0.4). The network was imported in TSV format into Cytoscape 3.10.3 for visualization and topological analysis. The built-in “Centiscape 2.0″tool in Cytoscape was used to calculate multiple topological parameters, including degree centrality, closeness centrality, and betweenness centrality, to assess node importance within the network. Finally, genes were ranked based on closeness centrality, with higher values indicating greater hub importance.

### Functional enrichment analysis

Gene Ontology (GO) systematically categorizes gene function into three classes: Cellular Component (CC), Molecular Function (MF), and Biological Process (BP). The Kyoto Encyclopedia of Genes and Genomes (KEGG) links genomic information with functional pathways at a systems level. In this study, the R package clusterProfiler was used to perform GO and KEGG functional enrichment analyses on the screened targets.

### Development and evaluation of a BaP-PCa risk model using machine learning

We employed a comprehensive machine learning framework to evaluate the prognostic performance of BaP-related gene signatures, systematically assessing 101 combinations derived from 10 algorithms: Random Forest (RF), Support Vector Machine with radial basis function kernel (SVM), eXtreme Gradient Boosting (XGBoost), LightGBM, Elastic Net (EN), Ridge Regression, Lasso, Decision Tree (DT), k-Nearest Neighbors (KNN), and Neural Networks with a single hidden layer (NN), covering regularization-based regression, tree-based ensemble methods, and kernel-based modeling strategies. The 101 combinations correspond to different hyperparameter configurations and feature selection strategies. For each algorithm, we performed hyperparameter optimization using grid search combined with 10-fold cross-validation on the TCGA-PRAD training set, strictly tuning within each fold to prevent data leakage. The final model for each algorithm was selected based on the average performance across all folds. To mitigate overfitting, we required the models to maintain consistent predictive performance across three independent cohorts, TCGA-PRAD, GSE116918, and GSE46602, retaining only those robust models with a concordance index (C-index) > 0.65 in all three cohorts. More detailed information on the model framework can be found in our previous publication (Zhang K. et al., 2025; Xu et al., 2024).

### Molecular docking and protein expression validation

Molecular docking was employed to elucidate the interaction mechanisms between BaP and core BaP-PCa target proteins. The molecular structure of BaP was obtained from the PubChem database, and the three-dimensional structures of the target proteins were sourced from the AlphaFold Protein Structure Database. AutoDockTools 1.5.7 was used for molecular and protein structure preparation and to perform docking simulations, predicting binding modes, binding affinity (expressed as binding free energy, ΔG), and potential functional impacts. A binding free energy lower than 0 kcal/mol indicates spontaneous binding, while an energy below −5.0 kcal/mol suggests a stable complex. Furthermore, to determine the protein expression levels of the core targets, the Human Protein Atlas (HPA) database was queried to examine the expression of core targets in PCa and normal prostate tissues.

### Tumor microenvironment analysis

The CIBERSORT algorithm (Newman et al., 2015) was used to quantify the infiltration levels of 22 immune cell types in PCa tissues. Patients in the TCGA-PRAD cohort were divided into high and low expression groups based on the median RRM2 expression value to characterize changes in the tumor microenvironment (TME) composition. Pearson correlation analysis was subsequently performed to validate the correlation between RRM2 expression and the infiltration levels of immunosuppressive cells (regulatory T cells and M2 macrophages). To confirm the reliability of the CIBERSORT analysis results, differences in immunosuppression-related indicators between subgroups were further compared.

### Pan-cancer analysis of the core protein RRM2

To further elucidate the key role of RRM2 in cancer development and progression, we investigated its pan-cancer expression patterns using the SangerBox platform and performed univariate Cox regression analysis to evaluate the association between RRM2 expression and progression-free survival (PFS) across different tumor types.

### Cell culture and intervention

Human prostate cancer cells (DU145 and PC3) were purchased from the Cell Bank of the Chinese Academy of Sciences. Cells were cultured in a constant temperature incubator at 37 °C with 5% CO_2_, following the supplier’s recommended protocol. The formulations and doses of BaP and baicalin were based on previously reported methods and concentrations (Nwagbara et al., 2007). In these experiments, BaP was used at a concentration of 10 μM. Baicalin concentrations were determined based on the IC50 values from the dose-response curves reported in the literature (Miocinovic et al., 2005), with 100 μM used for treating DU145 cells and 150 μM for PC3 cells.

### Western blot

Total protein was extracted from DU145 and PC3 cells using RIPA lysis buffer (Solarbio, China) supplemented with a protease inhibitor cocktail (Yeasen, China). Protein samples were separated by 10% SDS-polyacrylamide gel electrophoresis and subsequently transferred to PVDF membranes using a wet transfer method. The membranes were blocked with 5% skim milk at room temperature for 1 h, followed by incubation with an anti-RRM2 primary antibody (1:5000 dilution; catalog #11661-1-AP, Proteintech) at 4 °C overnight. After washing with TBST, the membranes were incubated with species-appropriate secondary antibodies at room temperature for 2 h.

### EdU proliferation assay

To evaluate the effects of BaP and baicalin on the proliferation of DU145 cells, the 5-ethynyl-2′-deoxyuridine (EdU) cell proliferation assay kit (Beyotime Biotechnology, Shanghai, China) was used. Treated and untreated cells were seeded in 24-well plates at a density of 3 × 10^3^ cells per well. After 24 h of culture, 20 μL of EdU working solution was added to each well and incubated at 37 °C for 2 h. Subsequently, cell fixation and staining were performed according to the manufacturer’s instructions. Finally, EdU-positive cells were observed and recorded using a fluorescence microscope to quantitatively analyze the cell proliferation rate.

### Wound healing assay

To assess the effect of BaP and baicalin on cell migration capacity, a wound healing assay was performed. Treated and untreated DU145 cells were seeded in 6-well plates and cultured until fully confluent. A straight scratch was made on the cell monolayer using a sterile 200 μL pipette tip, and detached cells were gently washed away with PBS, establishing a standardized *in vitro* wound healing model. To exclude the influence of cell proliferation on migration analysis, the medium was replaced with low-serum migration medium. Images of the same field were captured at 0 h and 48 h post-scratch using a phase-contrast microscope (×10 objective), and cell migration capacity was quantitatively evaluated by comparing changes in scratch width.

### Transwell invasion assay

To evaluate cell invasion capacity, Transwell® chambers (polycarbonate membrane, 8 μm pore size) were used. Treated and control cells were resuspended in serum-free medium and seeded into the upper chamber. RPMI-1640 medium containing 10% FBS was added to the lower chamber as a chemoattractant. After incubation at 37 °C with 5% CO_2_ for 24 h, cells that migrated to the lower surface of the membrane were fixed with 4% paraformaldehyde and stained with 0.1% crystal violet. Finally, five random fields per sample were photographed using an inverted microscope (×10 objective), and migrated cells were counted for quantitative analysis.

### Statistical analysis

All statistical analyses were performed using R software (version 4.5.0). The nonparametric Wilcoxon rank-sum test was used to assess differences in gene expression and immune cell infiltration levels. Statistical significance was defined as a two-sided P-value <0.05. Data visualization was performed using the R package “ggplot2″ and the Sangerbox platform. The primary endpoint of this prognostic analysis was progression-free survival (PFS), defined as the time from initial diagnosis to biochemical recurrence (BCR) or the last follow-up. The BCR mentioned in the text serves as the event definition criterion for PFS.

## Result

### Initial assessment of BaP toxicity

Toxicological profiling using the ProTox 3.0 database indicated that Benzo[a]pyrene (BaP) presents a significant toxicological risk, demonstrating particularly high endocrine disruption activity and carcinogenic potential. Specifically, BaP showed high prediction scores for carcinogenicity (0.88) and mutagenicity (0.89). For endocrine disruption, BaP had a high probability of interacting with key receptors, including the aryl hydrocarbon receptor (AhR, 0.96), androgen receptor (AR, 0.78), estrogen receptor alpha (ERα, 0.64), and aromatase (0.83), comprehensive details are provided in Supplementary Table S4. These statistically significant predictions confirm a moderate-to-high health risk for BaP and provide a crucial toxicological foundation for our subsequent investigation into its mechanisms.

### Identification of BaP-PCa targets

**FIGURE 1:**
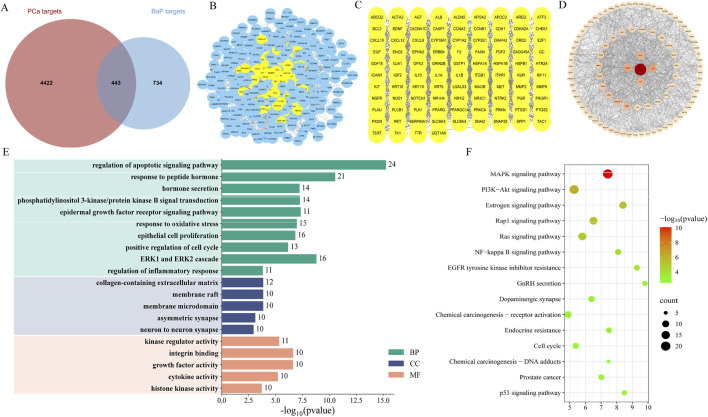
Identification of BaP-PCa overlapping targets and protein-protein interaction network analysis. **(A)** Venn diagram showing the intersection of BaP-associated genes (1,177 genes) and PCa-related differentially expressed genes (4,865 genes), identifying 443 overlapping genes as potential BaP-PCa targets. **(B)** Protein-protein interaction (PPI) network of the 443 overlapping targets, constructed using the STRING database with a confidence score threshold ≥0.4. **(C)** Core PPI network subnetwork extracted through topological analysis, highlighting hub proteins with high connectivity. **(D)** Topological analysis of the PPI network using Centiscape 2.0, ranking proteins by closeness centrality; higher values indicate greater hub importance. **(E)** Gene Ontology (GO) enrichment analysis of the 94 core genes, showing significantly enriched biological processes (BP), cellular components (CC), and molecular functions (MF). **(F)** Kyoto Encyclopedia of Genes and Genomes (KEGG) pathway enrichment analysis of the 94 core genes, revealing key signaling pathways potentially involved in BaP-mediated PCa progression.

We consolidated target predictions from multiple databases, identifying 1,177 unique genes associated with BaP (Supplementary Table S5). Differential expression analysis in prostate tumor tissues revealed 4,865 differentially expressed genes (Supplementary Table S5). A Venn diagram analysis identified 443 overlapping genes at the intersection of BaP-related and prostate cancer (PCa)-related genes (Figure 1A; Supplementary Table S5), suggesting their potential role as therapeutic targets in BaP-mediated PCa development and progression.

### Construction of the BaP-PCa PPI network

The 443 overlapping genes were used to construct a PPI network using the STRING database (confidence score ≥0.4). After removing unconnected nodes, the final network contained 421 proteins. Visualization with Cytoscape 3.10.3 and subsequent topological analysis identified 94 core proteins within the network. Five key hub proteins, ALB, BCL2, PPARG, AGT, and IL1B, were identified as having the highest connectivity (Figures 1B–D). This network visualization clarifies the interactions among key targets and provides a foundation for exploring the molecular mechanisms linking BaP to PCa.

### Functional enrichment analysis of BaP-PCa targets

GO enrichment analysis of the 94 core genes revealed their significant involvement in biological processes such as inflammatory response, cell proliferation, oxidative stress, and hormone secretion and transport (Figure 1E). KEGG pathway analysis indicated enrichment in several key pathways, including the MAPK, estrogen, Rap1, Ras, PI3K-Akt, and NF-kappa B signaling pathways, as well as pathways in chemical carcinogenesis (Figure 1F). These results suggest that BaP may promote PCa by disrupting inflammatory and cell proliferation processes, endocrine functions, and multiple established oncogenic pathways.

### Development and validation of a BaP-PCa prognostic model

**FIGURE 2:**
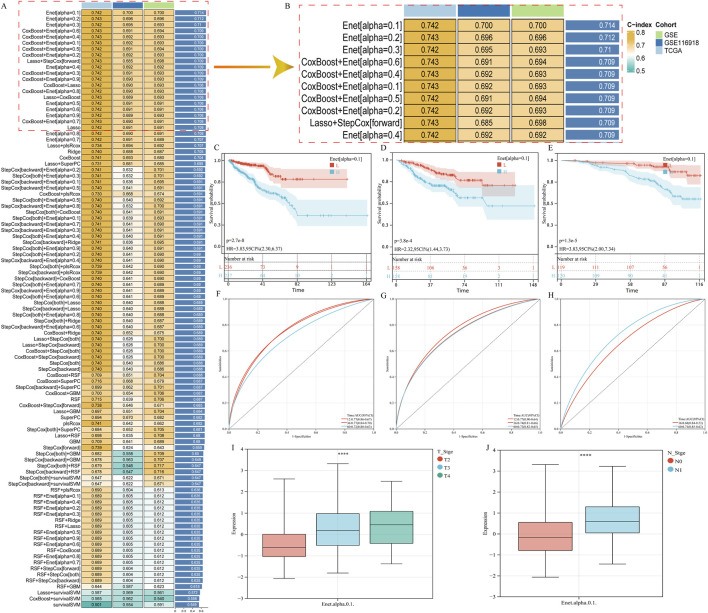
Development and validation of a BaP-PCa target-related prognostic model using machine learning. **(A)** Performance evaluation of 101 machine learning algorithm combinations for prognostic model construction. The Elastic Net model (α = 0.1) exhibited the highest average C-index (0.714) and was selected as the optimal model. **(B)** Coefficients of the 17 key genes included in the final Elastic Net model. **(C–E)** Kaplan-Meier survival curves for progression-free survival (PFS) comparing high-risk and low-risk groups stratified by median risk score in the TCGA-PRAD cohort **(C)** the combined GSE cohort (GSE21032 + GSE70770) **(D)** and the GSE116918 cohort **(E)**. Log-rank p-values are shown. **(F–H)** Time-dependent receiver operating characteristic (ROC) curves for predicting 1-, 3-, and 5-year PFS in the TCGA-PRAD cohort **(F)** the combined GSE cohort **(G)** and the GSE116918 cohort **(H)**. Area under the curve (AUC) values are indicated. **(I,J)** Correlation between risk score and clinical features, showing that higher risk scores are significantly associated with advanced pathological T stage **(I)** and higher Gleason score **(J)**. *p < 0.05, **p < 0.01, ***p < 0.001.

Using the 443 BaP-PCa targets, univariate Cox regression identified 34 genes significantly associated with PFS in the TCGA-PRAD and GSE cohorts (Supplementary Figure S1/S2). After systematically evaluating 101 algorithm combinations, the Elastic Net model [alpha = 0.1] demonstrated the best predictive performance. This optimal model incorporated 17 key genes (Figures 2A,B; Supplementary Table S6) and achieved a mean C-index of 0.714. Kaplan-Meier analysis confirmed that patients with high-risk scores had significantly shorter PFS in the TCGA-PRAD (Figure 2C), GSE (Figure 2D), and GSE116918 (Figure 2E) cohorts. Time-dependent ROC curves validated the model’s predictive accuracy, with 1-, 3-, and 5-year AUC values of 0.77, 0.77, and 0.72 in TCGA-PRAD (Figure 2F); 0.77, 0.74, and 0.73 in the GSE cohort (Figure 2G); and 0.68 and 0.74 for 3 and 5 years in GSE116918 (Figure 2H). Furthermore, the risk score was positively correlated with advanced clinical stage (Figures 2I,J). These findings highlight the prognostic value of this 17-gene signature and suggest these targets are critically involved in BaP-driven PCa progression.

### Molecular docking of BaP with the 17-gene signature

**FIGURE 3:**
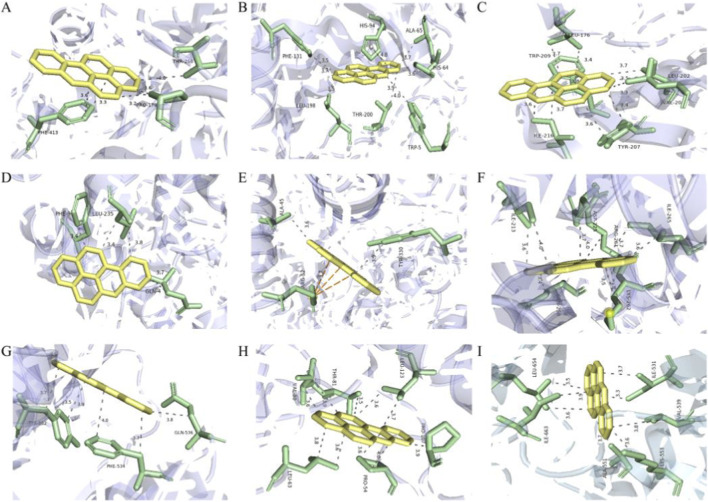
Molecular docking of BaP with nine candidate target proteins identified by machine learning. **(A)** ALDH1A3 (binding energy: −9.8 kcal/mol). **(B)** CDC20 (−9.2 kcal/mol). **(C)** CA2 (−7.2 kcal/mol). **(D)** EXO1 (−9.2 kcal/mol). **(E)** GRIN1 (−8.5 kcal/mol). **(F)** PDE4D (−8.4 kcal/mol). **(G)** PLK1 (−9.4 kcal/mol). **(H)** RRM2 (−11.5 kcal/mol). **(I)** TTK (−9.6 kcal/mol). For each protein, the left panel shows the overall docking conformation of BaP within the protein binding pocket, and the right panel shows detailed interactions including hydrogen bonds (green dashed lines) and hydrophobic interactions. Binding free energy (ΔG) was calculated using AutoDockTools 1.5.7; values < −5.0 kcal/mol indicate stable complex formation.

We used molecular docking to evaluate the binding between BaP and the core BaP-PCa proteins. After excluding proteins with unsuitable binding pockets or a binding energy (ΔG) of zero, ten proteins were identified as forming stable complexes with BaP (ΔG < −5 kcal/mol). These were: ALDH1A3 (−9.8 kcal/mol, Figure 3A), CDC20 (−9.2, Figure 3B), CA2 (−7.2, Figure 3C), EXO1 (−9.2, Figure 3D), GRIN1 (−8.5, Figure 3E), PDE4D (−8.4, Figure 3F), PLK1 (−9.4, Figure 3G), RRM2 (−11.5, Figure 3H), and TTK (−9.6, Figure 3I). Although FOXN3 also exhibited a favorable energy score (−8.7 kcal/mol), it was excluded due to the absence of stabilizing hydrogen bonds with BaP. These nine proteins that stably bind BaP *in silico* are proposed as candidate key targets that may mediate the potential effects of BaP on PCa, based on computational predictions.

### Prognostic value and expression of the 9 core targets

**FIGURE 4:**
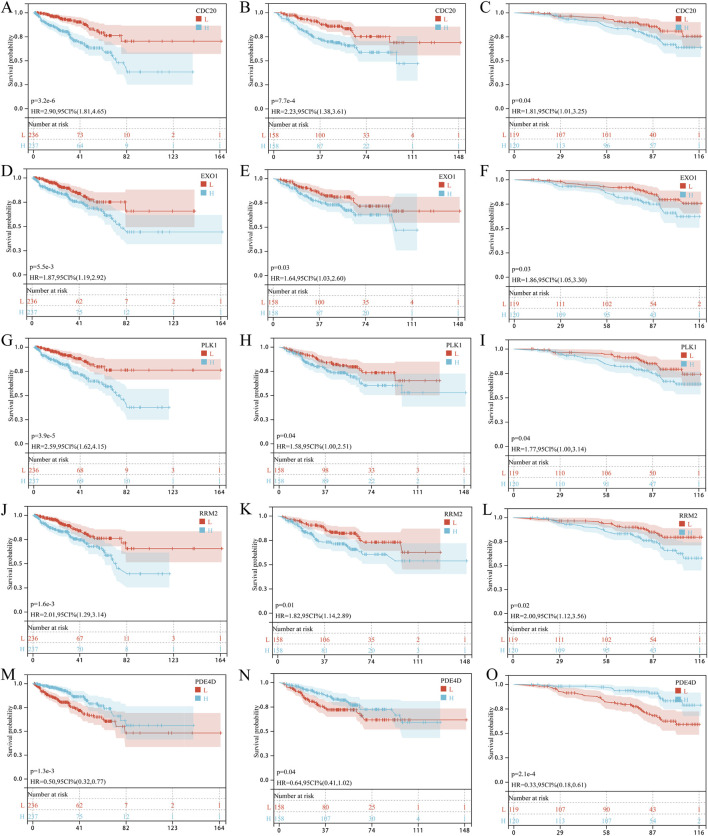
Prognostic significance of five core genes consistently associated with progression-free survival across three independent cohorts. **(A–C)** CDC20: Kaplan-Meier survival curves for PFS in the TCGA-PRAD cohort **(A)** the combined GSE cohort **(B)** and the GSE116918 cohort **(C)**. **(D–F)** EXO1: survival curves in TCGA-PRAD **(D)** GSE **(E)** and GSE116918 **(F)**. **(G–I)** PLK1: survival curves in TCGA-PRAD **(G)** GSE **(H)** and GSE116918 **(I)**. **(J–L)** RRM2: survival curves in TCGA-PRAD **(J)** GSE **(K)** and GSE116918 **(L)**. **(M–O)** PDE4D: survival curves in TCGA-PRAD **(M)** GSE **(N)** and GSE116918 **(O)**. Patients were stratified into high-expression (red) and low-expression (blue) groups based on median gene expression levels. Log-rank p-values are shown for each comparison. High expression of CDC20, EXO1, PLK1, and RRM2 was consistently associated with shorter PFS across all three cohorts, while high PDE4D expression was associated with longer PFS.

**FIGURE 5:**
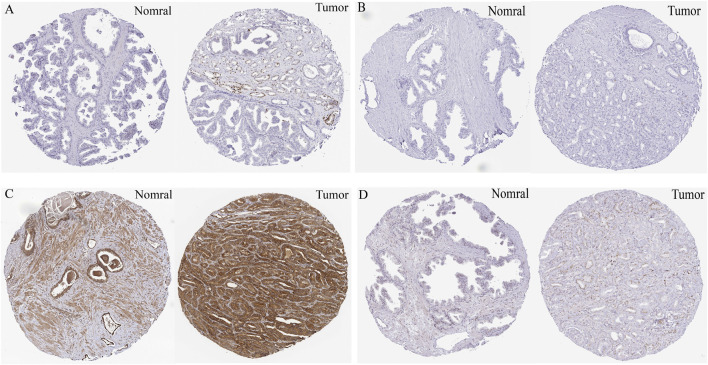
Protein expression validation of core genes in normal and prostate cancer tissues using the Human Protein Atlas (HPA) database. Representative immunohistochemistry (IHC) staining images from normal prostate tissue (left) and prostate cancer tissue (right) for **(A)** CDC20, **(B)** RRM2, **(C)** PDE4D, and **(D)** PLK1. Scale bars represent 100 μm. CDC20 and RRM2 show markedly increased staining intensity in tumor tissues compared to normal tissues, consistent with their mRNA expression patterns. PDE4D and PLK1 show comparable expression levels between normal and tumor tissues.

We next assessed the association between the 9 core targets and PCa prognosis using Kaplan-Meier survival analysis across three independent cohorts (TCGA-PRAD, GSE, and GSE116918). Only five targets were consistently associated with patient outcomes. High expression of CDC20, EXO1, PLK1, and RRM2 was correlated with shorter PFS in all three cohorts, while high PDE4D expression was associated with longer PFS (Figure 4). Analysis of the HPA database revealed that protein levels of CDC20 and RRM2 were significantly elevated in tumor tissues (Figures 5A,B; Supplementary Table S7). PLK1 and PDE4D were highly expressed in both normal and tumor tissues (Figures 5C,D), and EXO1 data were unavailable. Given that RRM2 exhibited the strongest predicted binding affinity with BaP in docking studies, was consistently associated with poor prognosis across all cohorts, and showed elevated expression at both the transcript and protein levels, we selected it as a candidate for further exploratory analysis.

### The impact of RRM2 on the PCa microenvironment

**FIGURE 6:**
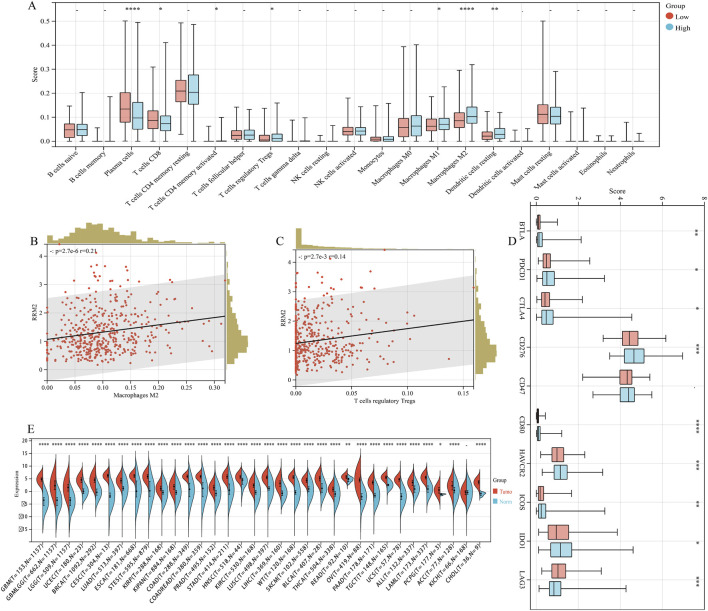
Association of RRM2 expression with tumor immune microenvironment and pan-cancer expression analysis. **(A)** Immune cell infiltration profiles in RRM2-high (n = 247) and RRM2-low (n = 247) groups from the TCGA-PRAD cohort, estimated using the CIBERSORT algorithm. Box plots show the relative abundance of 22 immune cell types. Statistical comparisons were performed using the Wilcoxon rank-sum test. **(B)** Pearson correlation analysis between RRM2 expression and regulatory T cell (Treg) infiltration (r = 0.14, p < 0.001). **(C)** Pearson correlation analysis between RRM2 expression and M2 macrophage infiltration (r = 0.21, p < 0.001). **(D)** Differential expression of immune checkpoint genes between RRM2-high and RRM2-low groups. Box plots show expression levels of 10 immune checkpoint molecules; 8 of 10 show significant upregulation in the RRM2-high group (*p < 0.05, **p < 0.01, ***p < 0.001). **(E)** Pan-cancer analysis of RRM2 expression across 33 TCGA cancer types. Box plots show RRM2 expression levels in tumor tissues (red) compared to matched normal tissues (blue) for each cancer type. Statistical significance: *p < 0.05, **p < 0.01, ***p < 0.001. ACC, adrenocortical carcinoma; BLCA, bladder urothelial carcinoma; BRCA, breast invasive carcinoma; etc.

Given prior evidence linking BaP to immunosuppression (Zhang Z. et al., 2025; Kang et al., 2022) and emerging studies implicating RRM2 in immune modulation (Tang et al., 2021; Li et al., 2022; Lee et al., 2023), we explored whether RRM2 expression is associated with immune infiltration patterns in PCa. Our CIBERSORT analysis revealed modest but statistically significant correlations between RRM2 expression and the infiltration levels of Tregs (r = 0.14, p < 0.001) and M2 macrophages (r = 0.21, p < 0.001) (Figures 6A-C). These observations suggest that RRM2-high tumors may exhibit a tendency toward an immunosuppressive microenvironment, although the modest correlation coefficients indicate that this association is subtle and likely context-dependent. Additionally, we observed upregulation of multiple immune checkpoint molecules in the RRM2-high group (Figure 6D). Collectively, these findings generate the hypothesis that RRM2 could be involved in immune modulation in PCa; however, experimental validation, including functional studies directly linking BaP exposure to RRM2-mediated immune remodeling, is required to establish a causal relationship.

### Pan-cancer analysis of RRM2

A pan-cancer analysis using the SangerBox database revealed that RRM2 is significantly overexpressed in nearly all cancer types examined (Figure 6E). Furthermore, high RRM2 expression was associated with shorter PFS in multiple cancers, including GBMLGG, KIPAN, KIRP, PRAD, and others (Supplementary Figure S3). These results underscore the broad and significant role of RRM2 in tumorigenesis and progression, positioning it as a potential key molecular target in BaP-driven carcinogenesis.

### Screening of potential natural product interventions

**FIGURE 7:**
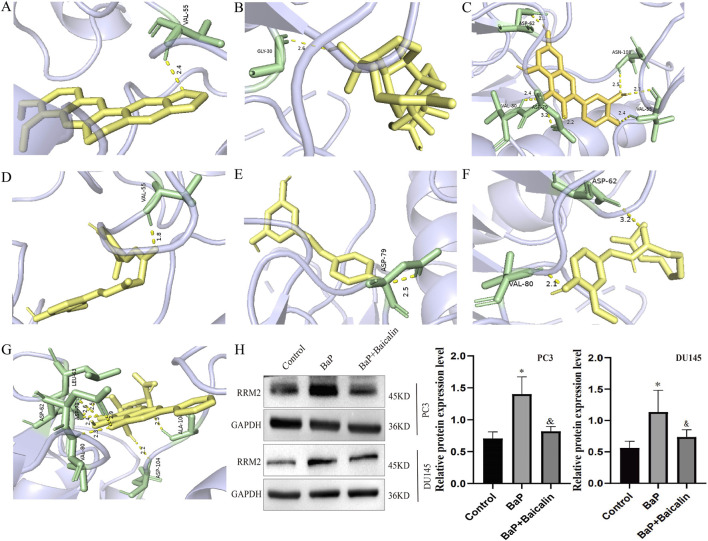
Molecular docking of seven natural bioactive compounds with RRM2 and experimental validation of RRM2 expression modulation. **(A–G)** Molecular docking results showing the binding conformations of natural compounds with RRM2: **(A)** Baicalin (binding energy: −10.8 kcal/mol), **(B)** Quercetin (−9.9 kcal/mol), **(C)** Berberine (−9.2 kcal/mol), **(D)** Curcumin (−8.5 kcal/mol), **(E)** Ginsenoside (−7.9 kcal/mol), **(F)** Resveratrol (−7.3 kcal/mol), **(G)** Capsaicin (−6.5 kcal/mol). For each compound, the left panel shows the overall docking conformation within the RRM2 binding pocket, and the right panel shows detailed molecular interactions including hydrogen bonds (green dashed lines). **(H)** Western blot analysis of RRM2 protein expression in DU145 cells under different treatment conditions. Cells were treated with vehicle control, BaP (10 μM), baicalin (100/150 μM), or combination of BaP and baicalin for 48 h β-actin was used as a loading control. Bar graph shows quantitative analysis of RRM2 protein levels normalized to β-actin, expressed as fold change relative to control. Data are presented as mean ± SD from three independent experiments. **p < 0.01, ***p < 0.001 compared to control; #p < 0.01 compared to BaP alone.

Given the increasing importance of natural products in anticancer drug discovery (Slika et al., 2022), we selected seven naturally occurring bioactive compounds: baicalin, ginsenoside, quercetin, curcumin, resveratrol, capsaicin, and berberine. The selection was based on the following criteria: (a) documented antitumor activity against prostate cancer as reported in the literature; (b) representation of diverse structural classes (including flavonoids, alkaloids, polyphenols, etc.) to evaluate the influence of structural variability on binding affinity; and (c) favorable safety profiles and promising potential for clinical application. Molecular docking analyses confirmed that all seven compounds bind stably to the RRM2 protein (Figures 7A–G). The calculated binding energies were as follows: baicalin (−10.8 kcal/mol), quercetin (−9.9 kcal/mol), berberine (−9.2 kcal/mol), curcumin (−8.5 kcal/mol), ginsenoside (−7.9 kcal/mol), resveratrol (−7.3 kcal/mol), and capsaicin (−6.5 kcal/mol). Given that baicalin exhibited the strongest binding affinity (lowest docking energy) and has been previously reported to possess therapeutic potential against prostate cancer, it was selected for subsequent analyses.

### *In vitro* effects of BaP and baicalin on PCa cells

**FIGURE 8:**
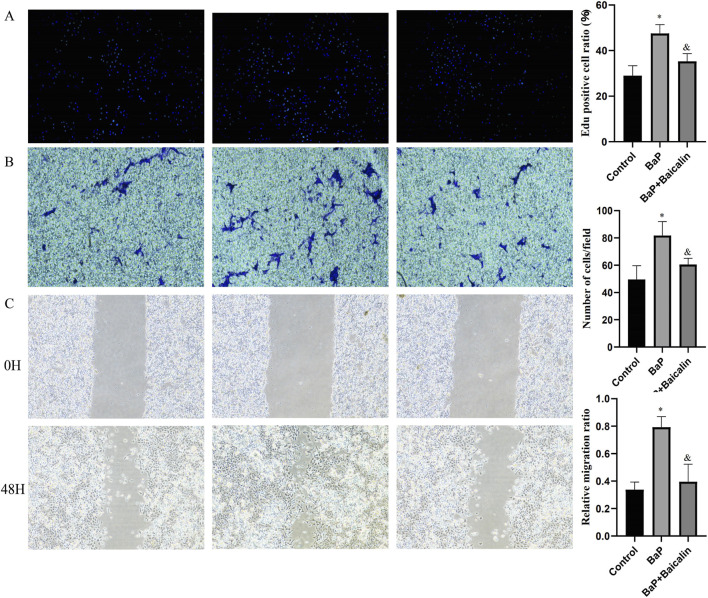
Effects of BaP and baicalin on prostate cancer cells. **(A)** Edu proliferation assay. **(B)** Transwell assay. **(C)** Wound healing assay. Scale bar, 100 μm. **p < 0.05 versus control group; ##p < 0.05 versus BaP-treated group.

Western blot analysis revealed that BaP treatment further increased RRM2 protein expression levels in PCa cells (DU145 and PC3) (Figure 7H). Subsequent functional assays demonstrated that BaP intervention significantly promoted PCa cell proliferation (EdU assay, Figure 8A), invasion (Transwell assay, Figure 8B), and migration (wound healing assay, Figure 8C). Conversely, baicalin intervention not only reversed the BaP-induced upregulation of RRM2 expression but also counteracted the BaP-mediated enhancement of proliferation, migration, and invasion in PCa cells. These findings further underscore that BaP may promote malignant progression of PCa through upregulating RRM2 expression, while baicalin may serve as a potential interventional agent against this effect.

## Discussion

BaP, a classic environmental pollutant from incomplete combustion, continues to pose a public health risk through long-term, low-dose exposure due to its environmental persistence, bioaccumulation, and established carcinogenic and endocrine-disrupting properties, despite regulatory controls (Huang et al., 2025; Hummel et al., 2018; Araki et al., 2024). Its link to hormone-dependent cancers like PCa is of growing concern (Zhang et al., 2016; Bukowska et al., 2022; Modica et al., 2023; Shi et al., 2017). However, significant gaps remain in understanding the mechanisms by which BaP may induce PCa initiation and progression. To address this knowledge gap, we employed a multidisciplinary strategy integrating bioinformatics, machine learning, molecular docking, and preliminary *in vitro* experiments to generate and explore hypotheses about these mechanisms and to identify candidate target genes. By constructing a multi-omics network connecting predicted BaP targets with PCa-associated genes, we identified potential core genes and their interactions, while also screening *in silico* for natural products that might counteract BaP toxicity. By constructing a multi-omics network linking BaP to PCa, we elucidated core genes and their interactions, while also screening for natural products that may counteract BaP toxicity. Our findings provide novel insights into the role of BaP in PCa and suggest potential preventive strategies for at-risk populations.

Our study began by confirming BaP’s carcinogenic and endocrine-disrupting potential through toxicological assessment. We then identified 443 BaP-PCa-associated genes via network toxicology. KEGG and GO enrichment analyses suggested that BaP promotes PCa by modulating biological processes such as inflammatory response, cell proliferation, hypoxia, and hormone metabolism, and by interfering with key signaling pathways including Ras, PPAR, and cAMP. Using a comprehensive machine-learning framework of 101 algorithm combinations, we developed a prognostic model based on 17 key BaP-PCa genes. This model demonstrated excellent performance in predicting biochemical recurrence, indicating the potential importance of these genes in BaP-mediated PCa progression. Subsequent molecular docking, Kaplan-Meier survival analysis, and validation with the HPA database pinpointed RRM2 as a central target: its expression was consistently associated with shorter progression-free survival across multiple independent cohorts, it exhibited the highest binding affinity for BaP, and its protein levels were significantly elevated in tumor tissues, consistent with transcriptomic data.

RRM2, a subunit of ribonucleotide reductase, is a key enzyme for *de novo* DNA synthesis and plays a central role in cell cycle regulation by maintaining the balance of dNTP production and DNA replication. Its expression is tightly regulated during the cell cycle, and its delayed degradation can lead to genomic instability (Zuo et al., 2022; Greene et al., 2020; Aye et al., 2015). RRM2 is overexpressed in many malignancies and is closely linked to tumorigenesis and progression (Zuo et al., 2022; Greene et al., 2020; Aye et al., 2015; Xiong et al., 2021; Du et al., 2024; Corrales-Guerrero et al., 2023). In PCa specifically, previous studies have highlighted its association with disease advancement, including metastasis, recurrence, and resistance to anti-androgen therapy (Cheng et al., 2023; Mazzu et al., 2019; Mazzu et al., 2020; Dong et al., 2023). Our findings align with this trend, showing RRM2 overexpression in PCa and many other cancers, correlated with shorter PFS. Notably, the potential connection between RRM2 and BaP has been largely overlooked, with no prior studies exploring RRM2’s role in BaP-related carcinogenesis. Our research begins to address this gap: molecular docking predicted a high binding affinity between BaP and RRM2, and Western blot analysis demonstrated that BaP treatment was associated with increased RRM2 protein levels in PCa cells under the tested conditions. These findings provide preliminary experimental evidence supporting the hypothesis that BaP may influence PCa cell behavior potentially through modulation of RRM2 expression, although further functional studies are needed to confirm this relationship.

Furthermore, emerging evidence links BaP to the formation of an immunosuppressive TME that facilitates PCa progression (Zhang Z. et al., 2025; Kang et al., 2022). The TME, composed of tumor cells and surrounding immune/stromal components, provides a critical niche for tumor survival and advancemen^t^ (Kang et al., 2022), and its reprogramming influences therapeutic response and clinical outcomes (Comito et al., 2019). Interestingly, recent studies also implicate RRM2 in shaping an immunosuppressive TME (Tang et al., 2021; Li et al., 2022; Lee et al., 2023). We therefore hypothesized that RRM2 participates in the BaP-mediated formation of an immunosuppressive niche in PCa. Our analysis of the PCa TME revealed that high RRM2 expression correlates with increased infiltration of Tregs and M2 macrophages—key immunosuppressive populations that drive tumor progression, immune evasion, angiogenesis, and therapy resistance. Additionally, high RRM2 expression was associated with the upregulation of multiple immune checkpoint molecules.

However, although our tumor microenvironment analysis is suggestive, it has important limitations. The correlations between RRM2 expression and immune cell infiltration, though statistically significant, were modest in strength, indicating that RRM2 is likely only one of multiple factors influencing immune composition. Importantly, our study lacks independent validation of immune infiltration patterns using complementary methods (e.g., immunohistochemistry or flow cytometry) and does not provide experimental evidence directly linking BaP exposure to RRM2-mediated immune remodeling. Therefore, the relationship between BaP, RRM2, and the immune microenvironment in PCa remains an intriguing hypothesis that warrants dedicated investigation in future studies employing appropriate *in vivo* models and immune profiling techniques. Furthermore, although RRM2 is universally overexpressed across various cancer types, whether its specific mechanisms of action in the context of BaP exposure are tissue-specific remains to be further elucidated. Future studies employing cross-cancer comparative analyses could explore the differential associations of RRM2 with BaP metabolic pathways, immune microenvironment, and clinical prognosis across distinct tumor types, thereby elucidating its potential tissue-dependent functions.

Finally, natural products, with their multi-target and multi-pathway pharmacological properties, are an important source for anti-cancer drug discovery (Slika et al., 2022). Their potential to mitigate health damage from environmental pollutants is also gaining attention (Watkins et al., 2007; Ghahri et al., 2024). Consequently, identifying natural compounds that antagonize BaP’s carcinogenic effects is highly relevant for developing protective strategies. In this study, we confirmed via molecular docking that seven natural products with established anti-tumor activity can bind to RRM2. Subsequent *in vitro* experiments demonstrated that baicalin effectively suppressed the BaP-induced upregulation of RRM2. This provides experimental support for the application of natural products in preventing and controlling endocrine-disruptor-related tumors.

Furthermore, it is worth discussing the concentration of BaP used in the *in vitro* experiments of this study. The selection of 10 μM BaP was based on previous reports (Nwagbara et al., 2007) and took into account both experimental feasibility and the need for mechanistic exploration. Notably, this concentration exceeds typical environmental exposure levels in the general population, warranting further clarification of its physiological relevance. Human exposure assessment data indicate that the daily intake of BaP in the general population ranges from 50 to 613 ng, while the cumulative dose in occupationally exposed individuals can reach up to 300 μg/m^3^·year (Huang et al., 2025; Hummel et al., 2018; Araki et al., 2024). As a lipophilic environmental pollutant, BaP tends to accumulate in adipose tissue and can be enriched through the food chain. Chronic low-dose exposure may lead to gradually increasing concentrations in target organs such as the prostate, potentially far exceeding free concentrations in the blood. However, data on the actual accumulated concentration of BaP in prostate tissue remain very limited. *In vitro* studies often employ relatively higher concentrations (e.g., 1–10 μM) to simulate the molecular events triggered by chronic long-term exposure within a short timeframe,a common strategy in environmental toxicology mechanism research. In the present study, 10 μM BaP effectively upregulated RRM2 expression and induced pro-tumorigenic phenotypes within 48 h, indicating that this concentration is suitable for revealing the potential molecular mechanisms of BaP. Nevertheless, future studies should integrate chronic low-dose exposure models, three-dimensional organoids, or animal models to more accurately simulate real-world exposure scenarios and validate whether the findings of this study hold true at lower concentrations.

Compared to previous studies, our work offers several methodological advancements. First, the integration of bioinformatics, machine learning, molecular docking, and *in vitro* validation creates a robust, multi-layered strategy that enhances the systematic rigor of our findings. Second, we not only identified RRM2 as a key player in BaP-mediated carcinogenesis but also were the first to demonstrate that a natural product, baicalin, can counteract the carcinogenic effects induced by BaP. These discoveries provide a scientific basis for dietary interventions, such as the consumption of specific natural active compounds, in high-risk populations.

Despite its strengths, this study has several limitations. First and foremost, it lacks direct validation using human cohorts with individual-level BaP exposure data. The association between BaP and PCa was inferred through bioinformatics predictions of target overlap and molecular docking, rather than from epidemiological cohorts stratified by environmental exposure levels. While the TCGA-PRAD and GEO cohorts used for prognostic modeling are invaluable, they do not contain information on patients’ BaP exposure history. Consequently, our findings represent molecular features associated with PCa aggressiveness that overlap with predicted BaP targets, but the direct link between environmental BaP exposure and these features in patients remains to be established. Second, the target identification primarily relies on bioinformatics predictions, which may be subject to algorithmic biases and confidence thresholds, potentially affecting the accuracy and comprehensiveness of the identified BaP targets. Third, the reliance on *in vitro* cell models limits our ability to recapitulate the complex pharmacokinetics (such as absorption, distribution, metabolism, and excretion) of the whole organism. Finally, although Western blotting confirmed changes in RRM2 expression, the upstream regulatory mechanisms and specific roles of downstream signaling pathways warrant further investigation through additional molecular experiments.

## Conclusion

In summary, this study employed an integrated bioinformatics approach, supplemented by preliminary *in vitro* validation, to propose a mechanistic model for the molecular link between BaP and PCa. Our findings suggest that BaP may influence PCa through multiple key targets and signaling pathways, with RRM2 identified as a candidate hub molecule within the predicted BaP-PCa interaction network. This work provides a theoretical framework for understanding the potential mechanisms of BaP in PCa, generates testable hypotheses, and highlights the potential role of natural bioactive products in preventing carcinogenesis induced by environmental pollutants. However, these findings require validation in future studies involving exposure-stratified human cohorts and functional experimental investigations.

## Data Availability

The datasets presented in this study can be found in online repositories. The names of the repository/repositories and accession number(s) can be found in the article/Supplementary Material.

## References

[B1] Araki S. Shimadera H. Chatani S. Kitayama K. Shima M. (2024). Long-term spatiotemporal variation of benzo[a]pyrene in Japan: significant decrease in ambient concentrations, human exposure, and health risk. Environ. Pollut. 360, 124650. 10.1016/j.envpol.2024.124650 39111529

[B2] Aye Y. Li M. Long M. J. Weiss R. S. (2015). Ribonucleotide reductase and cancer: biological mechanisms and targeted therapies. Oncogene 34 (16), 2011–2021. 10.1038/onc.2014.155 24909171

[B3] Bukowska B. Mokra K. Michałowicz J. (2022). Benzo[*a*]pyrene-Environmental occurrence, human exposure, and mechanisms of toxicity. Int. J. Mol. Sci. 23 (11), 6348. 10.3390/ijms23116348 35683027 PMC9181839

[B4] Cheng B. Li L. Wu Y. Luo T. Tang C. Wang Q. (2023). The key cellular senescence related molecule RRM2 regulates prostate cancer progression and resistance to docetaxel treatment. Cell Biosci. 13 (1), 211. 10.1186/s13578-023-01157-6 37968699 PMC10648385

[B5] Comito G. Iscaro A. Bacci M. Morandi A. Ippolito L. Parri M. (2019). Lactate modulates CD4^+^ T-cell polarization and induces an immunosuppressive environment, which sustains prostate carcinoma progression *via* TLR8/miR21 axis. Oncogene 38 (19), 3681–3695. 10.1038/s41388-019-0688-7 30664688

[B6] Corrales-Guerrero S. Cui T. Castro-Aceituno V. Yang L. Nair S. Feng H. (2023). Inhibition of RRM2 radiosensitizes glioblastoma and uncovers synthetic lethality in combination with targeting CHK1. Cancer Lett. 570, 216308. 10.1016/j.canlet.2023.216308 37482342

[B7] Cui H. Zhang W. Zhang L. Qu Y. Xu Z. Tan Z. (2024). Risk factors for prostate cancer: an umbrella review of prospective observational studies and mendelian randomization analyses. PLoS Med. 21 (3), e1004362. 10.1371/journal.pmed.1004362 38489391 PMC10980219

[B8] Dong Q. Qiu H. Piao C. Li Z. Cui X. (2023). LncRNA SNHG4 promotes prostate cancer cell survival and resistance to enzalutamide through a let-7a/RREB1 positive feedback loop and a ceRNA network. J. Exp. Clin. Cancer Res. 42 (1), 209. 10.1186/s13046-023-02774-2 37596700 PMC10436424

[B9] Du Z. Zhang Q. Xiang X. Li W. Yang Q. Yu H. (2024). RRM2 promotes liver metastasis of pancreatic cancer by stabilizing YBX1 and activating the TGF-Beta pathway. iScience 27 (10), 110864. 10.1016/j.isci.2024.110864 39398252 PMC11470400

[B10] Feijó M. Carvalho T. M. A. Fonseca L. R. S. Vaz C. V. Pereira B. J. Cavaco J. E. B. (2025). Endocrine-disrupting chemicals as prostate carcinogens. Nat. Rev. Urol. 22 (9), 609–631. 10.1038/s41585-025-01031-9 40379948

[B11] Ghahri A. Saboji M. Hatami H. Ranjbar A. Salimi A. Seydi E. (2024). Apigenin ameliorates petrol vapors-induced oxidative stress as occupational and environmental pollutants in rats: an *in vivo* study. Arch. Environ. Occup. Health 79 (3-4), 143–151. 10.1080/19338244.2024.2394418 39169800

[B12] Goldenberg S. L. Nir G. Salcudean S. E. (2019). A new era: artificial intelligence and machine learning in prostate cancer. Nat. Rev. Urol. 16 (7), 391–403. 10.1038/s41585-019-0193-3 31092914

[B13] Greene B. L. Kang G. Cui C. Bennati M. Nocera D. G. Drennan C. L. (2020). Ribonucleotide reductases: structure, chemistry, and metabolism suggest new therapeutic targets. Annu. Rev. Biochem. 89, 45–75. 10.1146/annurev-biochem-013118-111843 32569524 PMC7316142

[B14] Huang J. Yang Q. Zeng L. Deng K. (2025). Multi-omics network toxicology reveals the role of benzo[a]pyrene in ovarian cancer: integrating gut microbiota dynamics and Mendelian randomization. Ecotoxicol. Environ. Saf. 302, 118601. 10.1016/j.ecoenv.2025.118601 40602159

[B15] Hummel J. M. Madeen E. P. Siddens L. K. Uesugi S. L. McQuistan T. Anderson K. A. (2018). Pharmacokinetics of [C]-Benzo[a]pyrene (BaP) in humans: impact of Co-Administration of smoked salmon and BaP dietary restriction. Food Chem. Toxicol. 115, 136–147. 10.1016/j.fct.2018.03.003 29518434 PMC5935529

[B16] Kang J. La Manna F. Bonollo F. Sampson N. Alberts I. L. Mingels C. (2022). Tumor microenvironment mechanisms and bone metastatic disease progression of prostate cancer. Cancer Lett. 530, 156–169. 10.1016/j.canlet.2022.01.015 35051532

[B17] Lee S. K. Hwang Y. Han J. H. Haam S. Lee H. W. Koh Y. W. (2023). Characteristics of the immune microenvironment associated with RRM2 expression and its application to PD-L1/PD-1 inhibitors in lung adenocarcinoma. Am. J. Cancer Res. 13 (11), 5443–5454. 38058821 PMC10695782

[B18] Li Y. Fu W. Geng Z. Song Y. Yang X. (2022). A pan-cancer analysis of the oncogenic role of ribonucleotide reductase subunit M2 in human tumors. PeerJ 10, e14432. 10.7717/peerj.14432 36518297 PMC9744174

[B19] Li Y. Zhou T. Liu Z. Zhu X. Wu Q. Meng C. (2025). Air pollution and prostate cancer: unraveling the connection through network toxicology and machine learning. Ecotoxicol. Environ. Saf. 292, 117966. 10.1016/j.ecoenv.2025.117966 40022828

[B20] Mazzu Y. Z. Armenia J. Chakraborty G. Yoshikawa Y. Coggins S. A. Nandakumar S. (2019). A novel mechanism driving poor-prognosis prostate cancer: overexpression of the DNA repair gene, ribonucleotide reductase small subunit M2 (RRM2). Clin. Cancer Res. 25 (14), 4480–4492. 10.1158/1078-0432.CCR-18-4046 30996073 PMC6820162

[B21] Mazzu Y. Z. Armenia J. Nandakumar S. Chakraborty G. Yoshikawa Y. Jehane L. E. (2020). Ribonucleotide reductase small subunit M2 is a master driver of aggressive prostate cancer. Mol. Oncol. 14 (8), 1881–1897. 10.1002/1878-0261.12706 32385899 PMC7400792

[B22] Miocinovic R. McCabe N. P. Keck R. W. Jankun J. Hampton J. A. Selman S. H. (2005). *In vivo* and *in vitro* effect of baicalein on human prostate cancer cells. Int. J. Oncol. 26 (1), 241–246. 15586246

[B23] Modica R. Benevento E. Colao A. (2023). Endocrine-disrupting chemicals (EDCs) and cancer: new perspectives on an old relationship. J. Endocrinol. Invest 46 (4), 667–677. 10.1007/s40618-022-01983-4 36526827

[B24] Newman A. M. Liu C. L. Green M. R. Gentles A. J. Feng W. Xu Y. (2015). Robust enumeration of cell subsets from tissue expression profiles. Nat. Methods 12 (5), 453–457. 10.1038/nmeth.3337 25822800 PMC4739640

[B25] Ngiam K. Y. Khor I. W. (2019). Big data and machine learning algorithms for health-care delivery. Lancet Oncol. 20 (5), e262–e273. 10.1016/S1470-2045(19)30149-4 31044724

[B26] Nwagbara O. Darling-Reed S. F. Tucker A. Harris C. Abazinge M. Thomas R. D. (2007). Induction of cell death, DNA strand breaks, and cell cycle arrest in DU145 human prostate carcinoma cell line by benzo[a]pyrene and benzo[a]pyrene-7,8-diol-9,10-epoxide. Int. J. Environ. Res. Public Health 4 (1), 10–14. 10.3390/ijerph2007010002 17431309 PMC3719953

[B27] Patke R. Harris A. E. Woodcock C. L. Thompson R. Santos R. Kumari A. (2024). Epitranscriptomic mechanisms of androgen signalling and prostate cancer. Neoplasia 56, 101032. 10.1016/j.neo.2024.101032 39033689 PMC11295630

[B28] Raychaudhuri R. Lin D. W. Montgomery R. B. (2025). Prostate cancer: a review. JAMA 333 (16), 1433–1446. 10.1001/jama.2025.0228 40063046

[B29] Shi Q. Godschalk R. W. L. van Schooten F. J. (2017). Inflammation and the chemical carcinogen benzo[a]pyrene: partners in crime. Mutat. Res. Rev. Mutat. Res. 774, 12–24. 10.1016/j.mrrev.2017.08.003 29173495

[B30] Shi C. Cheng L. Yu Y. Chen S. Dai Y. Yang J. (2024). Multi-omics integration analysis: tools and applications in environmental toxicology. Environ. Pollut. 360, 124675. 10.1016/j.envpol.2024.124675 39103035

[B31] Siegel R. L. Giaquinto A. N. Jemal A. , and Cancer statistics (2024). J clin. 74(2):203. 10.3322/caac.21830 38230766

[B32] Slika H. Mansour H. Wehbe N. Nasser S. A. Iratni R. Nasrallah G. (2022). Therapeutic potential of flavonoids in cancer: ROS-Mediated mechanisms. Biomed. Pharmacother. 146, 112442. 10.1016/j.biopha.2021.112442 35062053

[B33] Tang B. Xu W. Wang Y. Zhu J. Wang H. Tu J. (2021). Identification of critical ferroptosis regulators in lung adenocarcinoma that RRM2 facilitates tumor immune infiltration by inhibiting ferroptotic death. Clin. Immunol. 232, 108872. 10.1016/j.clim.2021.108872 34648954

[B34] Wang G. Zhao D. Spring D. J. DePinho R. A. (2018). Genetics and biology of prostate cancer. Genes Dev. 32 (17-18), 1105–1140. 10.1101/gad.315739.118 30181359 PMC6120714

[B35] Watkins B. A. Hannon K. Ferruzzi M. Li Y. (2007). Dietary PUFA and flavonoids as deterrents for environmental pollutants. J. Nutr. Biochem. 18 (3), 196–205. 10.1016/j.jnutbio.2006.12.002 17296493

[B36] Xiong W. Zhang B. Yu H. Zhu L. Yi L. Jin X. (2021). RRM2 regulates sensitivity to sunitinib and PD-1 blockade in renal cancer by stabilizing ANXA1 and activating the AKT pathway. Adv. Sci. (Weinh) 8 (18), e2100881. 10.1002/advs.202100881 34319001 PMC8456228

[B37] Xu Q. T. Qiang J. K. Huang Z. Y. Jiang W. J. Cui X. M. Hu R. H. (2024). Integration of machine learning for developing a prognostic signature related to programmed cell death in colorectal cancer. Environ. Toxicol. 39 (5), 2908–2926. 10.1002/tox.24157 38299230

[B38] Zhang Y. Dong S. Wang H. Tao S. Kiyama R. (2016). Biological impact of environmental polycyclic aromatic hydrocarbons (ePAHs) as endocrine disruptors. Environ. Pollut. 213, 809–824. 10.1016/j.envpol.2016.03.050 27038213

[B39] Zhang K. Che B. Gao P. Li W. (2025a). A comprehensive analysis reveals the relationship between artificial sweeteners and prostate cancer. Front. Nutr. 12, 1646623. 10.3389/fnut.2025.1646623 41001131 PMC12457353

[B40] Zhang Z. Zhang W. Wang H. Chen H. Yu Y. (2025b). Immunosuppressive role of benzo[a]pyrene exposure in prostate cancer progression. J. Environ. Sci. (China) 156, 185–199. 10.1016/j.jes.2024.11.032 40412924

[B41] Zuo Z. Zhou Z. Chang Y. Liu Y. Shen Y. Li Q. (2022). Ribonucleotide reductase M2 (RRM2): regulation, function and targeting strategy in human cancer. Genes Dis. 11 (1), 218–233. 10.1016/j.gendis.2022.11.022 37588202 PMC10425756

